# Anti-Inflammatory and Antioxidant Properties of Casein Hydrolysate Produced Using High Hydrostatic Pressure Combined with Proteolytic Enzymes

**DOI:** 10.3390/molecules22040609

**Published:** 2017-04-10

**Authors:** Fatemeh Bamdad, Seulki Hazel Shin, Joo-Won Suh, Chamila Nimalaratne, Hoon Sunwoo

**Affiliations:** 1 Centre for Pharmacy & Health Research, Faculty of Pharmacy and Pharmaceutical Sciences, University of Alberta, 11361-87 Ave, Edmonton, AB T6G 2E1, Canada; bamdad@ualberta.ca (F.B.); sshin1@ualberta.ca (S.H.S.); nimalara@ualberta.ca (C.N.); 2 Center for Nutraceutical and Pharmaceutical Materials, Myongji University, Yongin, Gyeonggi 449-728, Korea

**Keywords:** casein, enzymatic hydrolysis, high hydrostatic pressure, antioxidant activity, anti-inflammation

## Abstract

Casein-derived peptides are shown to possess radical scavenging and metal chelating properties. The objective of this study was to evaluate novel anti-inflammatory properties of casein hydrolysates (CH) produced by an eco-friendly process that combines high hydrostatic pressure with enzymatic hydrolysis (HHP-EH). Casein was hydrolysed by different proteases, including flavourzyme (Fla), savinase (Sav), thermolysin (Ther), trypsin (Try), and elastase (Ela) at 0.1, 50, 100, and 200 MPa pressure levels under various enzyme-to-substrate ratios and incubation times. Casein hydrolysates were evaluated for the degree of hydrolysis (DH), molecular weight distribution patterns, and anti-inflammatory properties in chemical and cellular models. Hydrolysates produced using HHP-EH exhibited higher DH values and proportions of smaller peptides compared to atmospheric pressure-enzymatic hydrolysis (AP-EH). Among five enzymes, Fla-digested HHP-EH-CH (HHP-Fla-CH) showed significantly higher antioxidant properties than AP-Fla-CH. The anti-inflammatory properties of HHP-Fla-CH were also observed by significantly reduced nitric oxide and by the suppression of the synthesis of pro-inflammatory cytokines in lipopolysaccharide (LPS)-stimulated RAW 264.7 macrophage cells. Liquid chromatography with tandem mass spectrometry (LC-MS/MS) revealed that 59% of the amino acids of the peptides in HHP-Fla-CH were composed of proline, valine, and leucine, indicating the potential anti-inflammatory properties. In conclusion, the HHP-EH method provides a promising technology to produce bioactive peptides from casein in an eco-friendly process.

## 1. Introduction

It is well-recognized that food-derived peptides can exert beneficial biological activities in addition to their basic nutritional role [1]. These bioactive peptides are relatively short (typically 2–20 amino acids) and may possess antioxidant, anti-inflammatory, antihypertensive, antimicrobial, and anticancer properties that have a potential role in maintaining or promoting human health [2]. Both anti-inflammatory and antioxidant properties are two of the main topics considered for preventing chronic diseases such as cardiovascular disease and cancer due to oxidative stress, as well as abnormal inflammatory responses. The intake of natural food-derived peptides may delay the onset of diseases by reducing the oxidative damage and pro-inflammatory responses [3,4].

Among other animal-derived food sources, milk proteins are considered as a highly nutritious food component with a well-balanced essential amino acid composition, and have also been reported as a good source of bioactive components [5]. Approximately, casein accounts for 80% of the total milk proteins, which are mainly composed of αS1 (~40% of total casein), αS2 (~10% of total casein), β (~35% of total casein), and κ (~15% of total casein) casein sub-units [6]. Accordingly, casein phosphopeptides derived from gastrointestinal and commercial proteases may act as multifunctional bioactive peptides with radical scavenging and metal chelating properties, and may play a role in enhancing mineral bioavailability [7].

High hydrostatic pressure (HHP) has been used as an important food preservation technique to increase the shelf life of meat and vegetable products by inactivating harmful microbes [8,9]. Alternatively, the combined use of HHP and enzymatic hydrolysis (HHP-EH) to produce protein hydrolysates reduces the amount and reaction time of enzymes, resulting in increased hydrolysate yields [10,11]. Accelerated enzyme activity observed at a certain degree of HHP (50–200 MPa) can be a result of effective enzyme-substrate contact, achieved through an increased solute concentration due to the compressed solution and a higher rate of diffusion due to disintegrated cellular compartments [11,12]. In addition, partially unfolded proteins under HHP are more susceptible to enzyme cleavage [13,14]. Therefore, high hydrostatic pressure combined with enzymatic hydrolysis (HHP-EH) is an eco-friendly process compared to enzymatic hydrolysis under atmospheric pressure (AP-EH), because it uses a lower amount of enzymes and solvents. Several studies have reported the use of extremely high-pressure (400–800 MPa) as a pre-treatment before enzymatic hydrolysis [12,15,16], which may result in unacceptable protein denaturation. Previously, HHP-EH was used to obtain bioactive peptides from β-lactoglobulin [17], ovalbumin [18], chickpea protein [19], and pinto bean protein [20]. Recently, our lab developed a novel technology of HHP-EH to increase the degree of hydrolysis of hydrophobic proteins such as phosvitin [21].

As mentioned above, casein-derived peptides possess strong radical scavenging and metal chelating properties. A combined use of high hydrostatic pressure and enzymatic hydrolysis of casein may produce lower molecular weight peptides with potential antioxidant and anti-inflammatory properties. The main objectives of this study were to optimize the conditions for the hydrolysis of casein using the HHP-EH process to produce bioactive peptides and to assess the effect of HHP on the antioxidant and anti-inflammatory activities of the casein hydrolysates (CH). Various enzymes, enzyme-to-substrate (E:S) ratios, and pressure levels were used to hydrolyse casein, in order to determine the optimal conditions required to achieve the highest efficiency of enzymatic catalysis. The casein hydrolysates were then tested for antioxidant and anti-inflammatory properties, in chemical and cellular models. The effective peptide sequences were also characterized by LC–MS/MS, to identify the potential amino acids contributing to the antioxidant properties. The one-step hydrolysis technology developed in this study reduces the processing time, avoids the use of harsh chemicals or solvents, and conserves their bioactivities.

## 2. Results and Discussion

### 2.1. Effect of Different Enzymes and High Hydrostatic Pressure on Casein Hydrolysis

**Figure 1:**
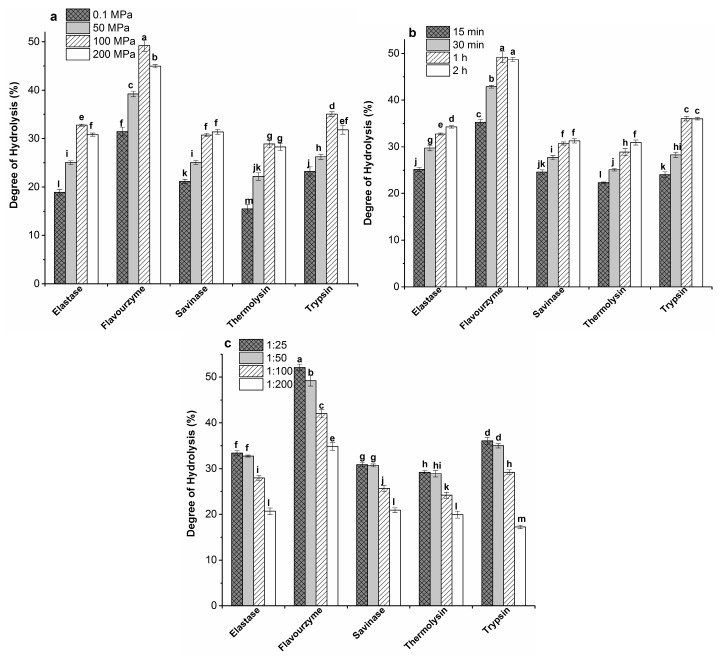
Degree of hydrolysis of casein hydrolysed under different conditions. (**a**) under atmospheric (0.1 MPa) and different high hydrostatic pressures (HHP, 50, 100, and 200 MPa) hydrolysed with different enzyme treatments at an enzyme-to-substrate (E:S) ratio of 1:50 for 1h; (**b**) different incubation times (15 and 30 min, 1 and 2 h) at an E:S ratio of 1:50 under 100 MPa; (**c**) at E: S ratios of 1:25, 1:50, 1:100, and 1:200 under 100 MPa for 1 h. Error bars are expressed as mean ± standard error with *n* ≥ 3. In each graph, the bars with different lowercase letters represent significant differences (*p <* 0.05) in degree of hydrolysis (DH) values.

Casein was hydrolysed by elastase (Ela), flavourzyme (Fla), savinase (Sav), thermolysin (Ther), trypsin (Try), under the optimum condition for each enzyme (Table 1). Figure 1a presents the effect of pressure levels (25, 50, 100, and 200 MPa) on the degree of hydrolysis (DH) (at E:S ratio of 1:50, hydrolysed for 1 h) of casein. Among the five enzymes used in our study, Fla and Try showed significantly higher DH values at HHP compared to AP (1.6- and 1.5-fold higher at 100 MPa than at AP (0.1 MPa), respectively, *p* < 0.05). Thus, these enzymes were used to prepare casein hydrolysates at an E:S ratio of 1:50, under 100 MPa pressure, and incubated for 1 h for the characterization studies.

In general, HHP (up to 100 MPa) changes the exposure of functional groups, which affects the protein volume (compressibility) and hydration shell surrounding the protein molecule, leading to a higher susceptibility of protein to enzymatic catalysis [10,22,23,24]. A higher efficiency of cellulase and β-amylase under 100 MPa was observed in the extraction of saponins from ginseng root (1.5- and 1.4-fold increase in saponin yield, respectively) compared to the AP condition [10]. Similar results were also reported by Garcia-Mora et al. [25] in the HHP assisted proteolysis of lentil proteins. They observed a significant increase in the short peptide content of hydrolysates obtained by commercial proteases. Higher pressure levels up to 150 MPa may disrupt larger protein aggregations and thereby increase the substrate accessibility, resulting in increased enzyme efficiency [26]. The pressure levels exceeding 150 MPa may cause the re-association of dissociated aggregates and enzyme inactivation [26,27]; however, at higher pressure levels (>200 MPa), the dissociation is irreversible and leads to smaller caseinate particles [28]. The disruption of electrostatic bridges leaves more charged groups on protein chains that attract water molecules. The diffusion of water to the protein cavities leads to a higher rate of mass transfer and efficient protein-enzyme contact [16].

### 2.2. Effect of Incubation Time on Casein Hydrolysis

Optimum casein hydrolysis at 100 MPa was evaluated by performing a series of experiments at different incubation times (15 min, 30 min, 1 h, and 2 h) and an E:S ratio of 1:50. Hydrolysis for a longer period resulted in a significant increase in the DH values in all samples compared to 15 min hydrolysis (Figure 1b). Hydrolysis for 2 h did not further increase the DH compared to 1 h in Fla, Sav, and Try-hydrolysed samples. This is in agreement with the previous report, which stated that the DH of Try hydrolysates of casein increases rapidly in the initial 20 min, reaching a plateau after 45 min, before remaining constant for the rest of the hydrolysis [29].

### 2.3. Effect of Enzyme-to-Substrate (E:S) Ratio on Casein Hydrolysis

Optimum casein hydrolysis at 100 MPa was evaluated at different E:S ratios (1:25, 50, 100, and 200) for 1 h. A higher amount of enzymes (E:S ratios of 1:25, 1:50) led to a higher DH compared to an E:S ratio of 1:200 (Figure 1c). In other words, with increased amounts of substrate in the mixture, the efficiency of the enzymes decreased. For four out of five of the enzymes used in the study (Ela, Sav, Ther, and Try), both the E:S ratios of 1:25 and 1:50 yielded similar DH values. Therefore, an E:S ratio of 1:50 was selected for further experiments, to reduce the cost and the usage of excess enzymes.

### 2.4. Molecular Weight Distribution of CH

**Figure 2:**
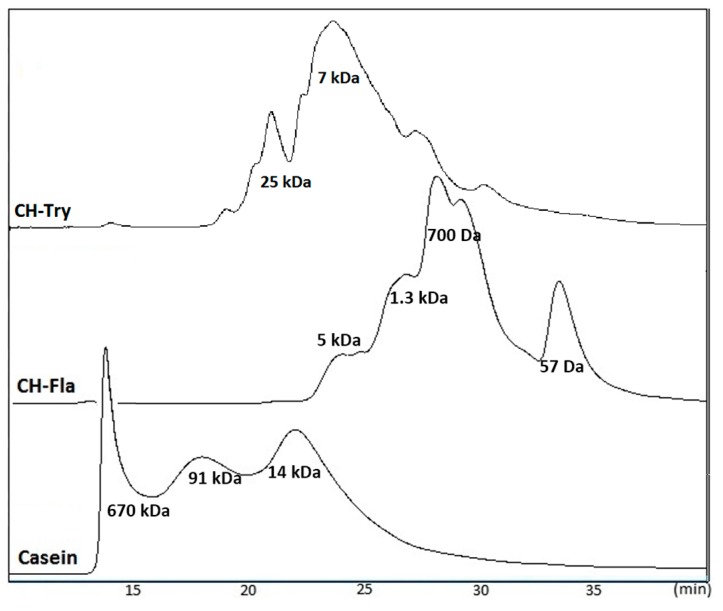
Size exclusion chromatograms of casein hydrolysates using different enzyme treatments at an E:S ratio of 1:50 under 100 MPa for 1 h.

Figure 2 illustrates the size exclusion chromatograms of intact casein and casein hydrolysates produced by Fla and Try. The intact casein chromatogram showed three peaks at 14, 18, and 22 min. The first and second peaks represent oligomers of casein molecules as their MW (670 and 91 kDa) are larger than individual caseins (MW range: 19–25 kDa). Enzymatic hydrolysis caused a pronounced shift of the peaks towards a lower MW range. Fla completely digested the intact casein peaks and produced short peptides with an MW value of less than 3 kDa. However, Try resulted in the partial hydrolysis of casein, which produced two peaks corresponding to 25 and 7 kDa peptides.

Matrix-assisted laser desorption/ionization time-of-flight (MALDI–TOF) mass spectra of samples showed that the MW of short peptides presented a wide range of values (500 to >2000 Da). Table 2 summarizes the relative area under the peaks categorized in the three MW ranges (500–1000, 1000–2000, and >2000 Da). It should be noted that, for a better accuracy and to avoid the background noises, only the peptides from 500 to 5000 Da were detected in the MALDI–TOF analysis. Therefore, the peptides smaller than 500 Da are not included in this peptide distribution. Fla-treated samples showed a very high content of 1–2 kDa peptides after digestion (Table 2). Moreover, remarkable increases occurred in the 500–1000 Da fraction under the HHP condition, especially in the Try sample. The Try-digested sample had higher proportions of 1–2 kDa and >2 kDa, and the relative area of the small peptide fraction (<1 kDa) increased to 26.5% under HHP conditions, compared to the 4.1% value which was obtained under AP digestion. Garcia-Mora et al. also observed a similar trend of the percentage distribution of peptide masses for HHP (at 300 MPa) and AP hydrolysis [25].

In a previous report, Morato et al. [30] studied the optimal conditions for the enzymatic hydrolysis of casein with a higher di- and tri-peptide content, to be used in dietary supplements. They obtained a hydrolysate containing only 14% of peptides and a molecular mass higher than 800 Da with a 4% E:S ratio, which is equivalent to 1:25, using subtilisin for 5 h hydrolysis. Fla is a commercial mixture of endo- and exo-peptidases obtained from a fungus (*Aspergillus oryzae*) and has been used to obtain low molecular weight peptides [31]. Rossini et al. (2009) also showed that casein hydrolysed by Fla resulted in a higher concentration of free amino acids and low molecular weight peptides, and also exhibited greater antioxidant properties [31]. In the current work, the use of a high hydrostatic pressure during casein hydrolysis by Fla resulted in a hydrolysate with significantly higher antioxidant properties and degree of hydrolysis compared to the hydrolysate obtained under atmospheric pressure. This study also demonstrated the anti-inflammatory properties of HHP-Fla-CH through its ability to suppress the NO production and synthesis of pro-inflammatory cytokines in lipopolysaccharide (LPS)-stimulated RAW 264.7 macrophage cells.

### 2.5. Antioxidant Capacity of CH

**Figure 3:**
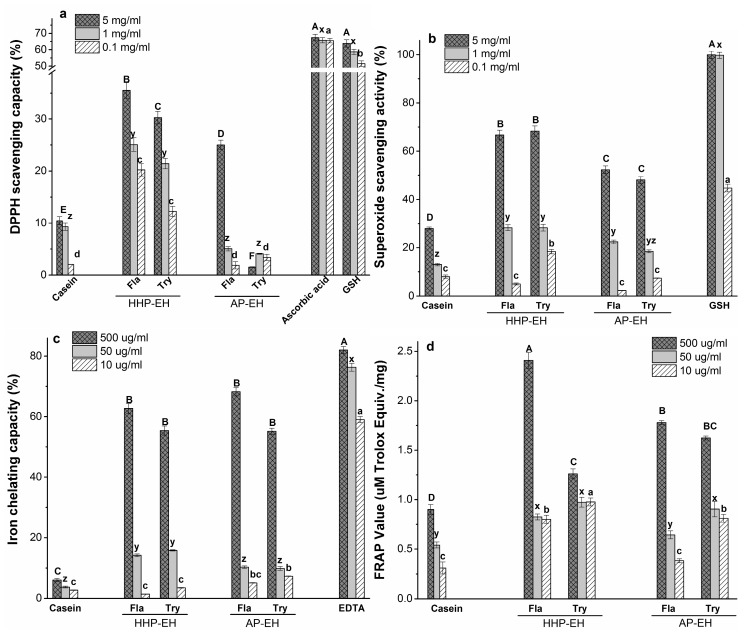
1,1-Diphenyl-2-picryl Hydrazyl (DPPH) scavenging capacity (**a**); Superoxide radical scavenging activity (**b**); Iron chelation capacity (**c**) and **f**erric **r**educing **a**ntioxidant **p**ower (FRAP) values (**d**) of intact casein and casein hydrolysates at different concentrations produced by Fla and Try under both HHP and atmospheric conditions. Error bars are expressed as mean ± standard error with *n* ≥ 3 (*p* < 0.05). For each concentration level, different letters indicate statistically significant differences (*p* < 0.05) among samples.

The antioxidant activity of hydrolysates may be attributed to the radical scavenging properties or the metal chelating capacity of peptides, or a combined effect of both. In this study, to better understand the antioxidant capacity of casein hydrolysates, we used 1,1-diphenyl-2-picryl Hydrazyl (DPPH) and superoxide scavenging assays to determine the radical scavenging capacity. For the metal chelating ability, iron chelating and ferric reducing antioxidant power (FRAP) assays were used. The enzymatic hydrolysis of casein under AP and HHP improved the antioxidant properties of CH (Figure 3).

#### 2.5.1. 1,1-Diphenyl-2-picryl Hydrazyl (DPPH) Radical Scavenging Capacity

DPPH is a relatively stable free radical, which is widely used for the in vitro evaluation of antioxidant compounds [32]. Free radicals are stabilized by hydrogen or electron donations from antioxidant peptides, leading to stable non-reactive compounds which cannot trigger the oxidation chain reactions [33]. As presented in Figure 3a, CH showed a dose-dependent DPPH scavenging capacity. Fla- and Try-CH exhibited a significantly higher (*p* < 0.05) scavenging capacity under HHP compared to AP. When considering that DPPH is a hydrophobic free radical, the more hydrophobic peptides that are present in the hydrolysate, the higher the expected DPPH scavenging activity will be [34]. The well-known oxidant scavengers such as ascorbic acid and glutathione (GSH) were used in the assay as positive controls. Although CHs have a lower scavenging capacity than ascorbic acid and glutathione, these peptides are considered to be safe to add to functional foods or cosmetic products. Additionally, as reviewed by Phelan et al. (2009), there is no maximum tolerated dose suggested for casein-derived peptides and some studies have demonstrated that the oral supplementation of casein-derived peptides in rats showed no adverse effects, even at doses as high as 40 mg per kg bodyweight per day [35,36].

#### 2.5.2. Superoxide Radical Scavenging Activity

Superoxide anion radicals (O_2_^•−^) can generate strong oxidants such as hydrogen peroxide and hydroxyl radicals in biological systems [37]. In the superoxide scavenging assay (Figure 3b), all CHs showed dose-dependent superoxide scavenging activity. Compared to casein, CH showed significantly higher activity at 5 and 1 mg/mL concentration levels. However, for both Fla and Try hydrolysates, significant differences between HHP-EH and AP-EH were only observed at a 5 mg/mL concentration level. Enzymatic hydrolysis increases the exposure of tyrosine, proline, glutamic acid, and leucine that have shown strong superoxide radical scavenging activity [38]. The most important factor in the scavenging superoxide radical is the peptide sequence, rather than the peptide size or conformation.

#### 2.5.3. Iron Chelating Activity

Iron and copper are the most important pro-oxidant metal ions. They facilitate oxidation reactions by catalysing hydroperoxide decomposition that results in hydroxyl radicals as the most reactive oxygen species [39,40]. Therefore, metal ion chelation plays an important role in the antioxidant mechanism of the peptides. Furthermore, recent evidence has shown that long chain peptides can entrap the metal ions in a cage-like structure [39]. The CH showed dose-dependent activity with the highest iron chelating activity (55–68%) being viewed at 500 μg/mL, which is comparable to ethylenediaminetetraacetic acid (EDTA) (60% at 10 μg/mL, *p* < 0.05) (Figure 3c). There are no differences in the iron chelating capacity of the HHP- and AP-EH samples, or among the Fla- and Try-CH at a higher concentration level (500 µg/mL).

#### 2.5.4. Reducing Capacity (FRAP assay)

A FRAP assay measures the electron-donor capacity of antioxidant compounds. In this assay, Fe^3+^ is reduced to Fe^2+^ by electron-donation from electron-rich amino acid side chains [41]. As presented in Figure 3d, at a 500 μg/mL concentration of peptides, only the HHP-Fla-CH sample showed a significantly higher FRAP value than AP compared with Try-CH (*p* < 0.05).

Possibly, smaller peptides could exhibit better reducing activity towards ferric ions, and suppress their pro-oxidant effect. Small peptides have a higher charge density (charge-to-mass ratio) than larger peptides, due to their more exposed electron-rich side chains. Similar results were observed by Lin et al. when studying the reducing capacity of small peptides (<1 kDa) obtained from egg white hydrolysates [42].

### 2.6. Anti-Inflammatory Properties of CH

#### 2.6.1. Cell Viability Assay

**Figure 4:**
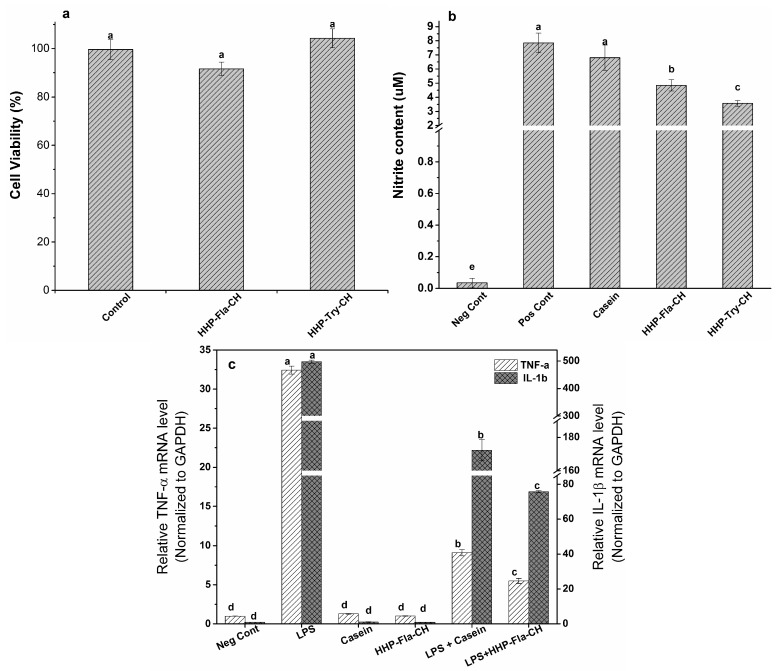
(**a**) Viability of RAW 264.7 cells; (**b**) Nitric oxide content of RAW 264.7 cell supernatant in the presence of intact casein or casein hydrolysates (at 1 mg/mL) produced under the HHP condition and (**c**) mRNA expression of pro-inflammatory cytokines (TNF-α and IL-1β) relative to glyceraldehyde 3-phosphate dehydrogenase (GAPDH) (∆∆C_t_) in LPS-stimulated RAW 264.7 macrophage cells in presence of intact casein (10 μg/mL) or Fla-hydrolysed casein (10 μg/mL). Values with different lower case letters are significantly (*p* < 0.05) different. Error bars are expressed as mean ± standard error with *n* ≥ 3 (*p* < 0.05).

The RAW 264.7 cell viability was evaluated to demonstrate the effect of CH on macrophage proliferation. The results (Figure 4a) showed that cells treated with all HHP-EH-CH (1 mg/mL) had >90% viability, indicating that CH did not influence RAW cell proliferation and thus, may be considered safe to the cells. It was previously demonstrated that whey protein hydrolysates produced under high pressure are well-tolerated (over 80% viability) by epithelial cells at peptide concentrations up to 1 mg/mL [43].

After 24 h incubation, both HHP- and AP-EH-CH did not show significant variations in cell viability. The detailed mechanisms of the uptake of peptides and the effect of peptides on cellular metabolism requires further investigation to ensure the safe use of peptides.

#### 2.6.2. Determination of NO Production by Macrophage Cells

Antioxidant peptides can reduce oxidative signaling, which is a part of inflammatory signaling cascades [43]. Oxidative stress stimulates the production of pro-inflammatory cytokines [44]. Nitric oxide is an important mediator of inflammatory processes. The overproduction of NO is associated with diabetes mellitus, neurodegenerative disorders, and other inflammatory conditions [45]. Therefore, in our study, the ability of HHP-EH-CH to attenuate NO production was estimated using LPS-induced macrophage cells (Figure 4b). The content of NO in a RAW 264.7 cell supernatant was evaluated after pre-incubation with HHP-EH-CH before LPS treatment (Figure 4b). The negative control showed a minimal content of NO (0.03 μM NO in cell supernatant), while LPS significantly stimulated NO production up to 7.8 μM NO. Pre-incubation with HHP-EH-CH significantly (*p* < 0.05) suppressed NO production to 3.5–6.5 μM NO, while non-hydrolysed casein showed no effect on NO production. Although both HHP-Fla-CH and HHP-Try-CH significantly reduced the production of NO, HHP-Try-CH was the most efficient in the down-regulation of NO production in macrophage cells.

In a previous study, Alcalase-hydrolysed soy protein showed an 18–35% inhibition in NO production in LPS-induced macrophage cells [46]. Different mechanisms have been proposed for the inhibition of LPS-induced effects on macrophage cell lines by proteins and peptides. Binding to the lipid A moiety of LPS and interference with the LPS-CD14 interaction by competence with the LPS-binding protein have been suggested [47]. Other factors, such as the amino acid sequence, also influence the cell internalization of peptides, which may change their inhibitory effect [48].

#### 2.6.3. Gene Expression of Pro-Inflammatory Cytokines in LPS-Stimulated Macrophages

Pro-inflammatory cytokine gene expression in macrophage cells was analysed to confirm the anti-inflammatory effect of HHP-Fla-CH. The mRNA level of pro-inflammatory cytokines (tumor necrosis factor (TNF-α) and interleukin (IL)1β (IL-1β)) increased in LPS-stimulated cells (Figure 4c). Without LPS-stimulation, the HHP-Fla-CH showed a minor effect on gene expression of TNF-α and IL-1β. Exposure to LPS strongly induced *TNF-α* and *IL-1β* gene expression by 32 and 500-times, respectively. The effect of HHP-Fla-CH on *TNF-α* and *IL-1β* gene expression was significantly higher (*p* < 0.05) than intact casein, and reduced the *TNF-α* and *IL-1 β* gene expression by 83 and 85%, respectively. The present data demonstrated that HHP-Fla-CH exhibited a remarkable inhibitory effect on *TNF-α* and *IL-1β* gene expression (*p* < 0.05).

### 2.7. Peptide Sequencing of the Most Potent CH 

HHP-Fla-CH was selected as the most potent sample for peptide sequencing due to the higher degree of hydrolysis, and high antioxidant and anti-inflammatory properties. A total of nine peptides characterized by the MS/MS and NCBI protein database search are shown in Table 3. Peptides with 6–14 amino acids ranging from 590 to 1500 Da mainly contain hydrophobic residues, making up about 59% of the sequence. The majority of antioxidant peptides have a molecular weight range of 500–1800 Da [49]. Peptide identification by LC–MS/MS indicated that the main source of milk-derived antioxidant peptides is β-casein, which contains the most hydrophobic residues. A high content of hydrophobic residues such as alanine, valine, leucine, and isoleucine shows a positive influence on scavenging free radicals [50]. Antioxidant peptides isolated from squid skin gelatin contained proline, alanine, valine, and leucine, which contribute the most to antioxidant activity [51]. All of the peptides identified in HHP-Fla-CH have more than one proline residue in their sequence. Proline has an electron-rich nitrogen-containing pyrrolidone ring that stabilizes the radical peptide formed after electron donation [52]. Proline-rich peptides are identified as multifunctional, with antioxidant, antimicrobial, and immunomodulatory properties, and thus may play a promising role in peptide-based food/supplement products [53]. Several studies have characterized antioxidant peptides with the amino acid sequence YFYPEL from αS1 casein [38], and QKALNEINQF, TKKTKLTEEEKNRL [54], FALPQYLK, and PYVRYL [55] from αS2 casein. The antioxidant properties of these peptides were mainly attributed to the presence of aromatic amino acids such as tyrosine and phenylalanine [55]. Among the identified sequences, five peptides have glutamic acid and glutamine as the terminal or penultimate amino acids, which can act as redox-active residues [52,56]. Therefore, our findings suggest that a proper balance between hydrophobic and polar amino acids may positively contribute to the antioxidant activity of casein peptides.

## 3. Materials and Methods

### 3.1. Materials

Sodium caseinate powder from bovine milk, flavourzyme (Fla), savinase (Sav), thermolysin (Ther), and phosvitin were purchased from Sigma-Aldrich (Oakville, ON, Canada). Trypsin (Try) was obtained from Thermo Fisher Scientific (Waltham, MA, USA). Elastase (Ela) was purchased from MP Biomedicals (Santa Ana, CA, USA). The mouse macrophage cell line, RAW 264.7, was obtained from the American Type Culture Collection (Rockville, MD, USA) and cultured in Dulbecco’s modified Eagle’s medium supplemented with 10% fetal bovine serum and penicillin/streptomycin/fungizone. LPS from *E. coli* 0111:B4 was from Sigma-Aldrich. Cell culture reagents (DMEM, FBS, Penicillin Streptomycin Glutamine, and Dulbecco’s Phosphated Buffered Saline), Ultrapure Distilled Water, and the TRIzol reagent were purchased from Invitrogen (Carlsbad, CA, USA). 5× All-In-One RT MasterMix and EvaGreen 2× qPCR MasterMix-Low ROX were from Applied Biological Materials (Richmond, BC, Canada). All primers were ordered from IDT (Coralville, IA, USA). All other chemicals and reagents were of analytical grade and purchased from commercial sources.

### 3.2. Apparatus

A portable high hydrostatic pressure instrument (TFS-2L, Toyo-Koatsu, Hiroshima, Japan) was used for the HHP-EH process. The vessel was filled with ultrapure water as a fluid of low compressibility and the internal pressure reached 50 and 100 MPa in about 1 and 2 min, respectively. The HHP instrument was designed with a fast decompression of the vessel for accurate pressure control. The protein-enzymes mixture was kept in a sealed pouch during the treatment, and the internal temperature of the vessel was automatically controlled by the computerized automatic pressure and temperature controlling system during the operation.

### 3.3. Enzymatic Hydrolysis of Casein

Casein was hydrolysed by Ela, Fla, Sav, Ther, and Try. Briefly, a caseinate solution (10 mg protein/mL distiller water) was mixed with each enzyme at four different enzyme:substrate (E:S) ratios (1:25, 1:50, 1:100, and 1:200 *w*/*w*). The pH of the mixtures was adjusted by the addition of 0.1 M HCl or NaOH, according to the optimum pH for each enzyme (Table 1). Each mixture was then placed in the HHP-EH chamber set at pressures of 50, 100, and 200 MPa for 15, 30, 60, and 120 min at the enzyme’s optimum temperature. Casein solution was also hydrolysed under atmospheric pressure (AP, 0.1 MPa) for comparison. The above procedures were performed in the absence of enzymes to act as experimental controls. After hydrolysis, the samples were heated at 100 °C for 10 min to inactivate the enzyme and were then centrifuged (4000*× g*, 30 min) to remove the insoluble parts. The supernatants were stored at −20 °C prior to analysis. The protein content of the casein samples was determined by the Bradford method using the Bio-Rad Proteins Assay kit (Bio-Rad, Mississauga, ON, Canada).

### 3.4. Determination of Degree of Hydrolysis (DH)

The DH was determined by the quantitative determination of the free amino groups released during hydrolysis, using an *Ο*-phthaldialdehyde (OPA) fluorometric assay [57]. The OPA solution (200 µL) was added to 20 µL of standard, samples, and control within the wells of a black microplate. The fluorometer readings were performed using a Synergy H1 Multi-Mode Reader (BioTek, Winooski, VT, USA) at excitation and emission wavelengths of 350 and 450 nm, respectively. The readings were corrected with unhydrolyzed casein to eliminate the effect of terminal amino groups. Deionized water was used as the control and l-serine was used as a standard. The DH was calculated by the following equation:

DH= (*h*/*h_tot_*) × 100 (1)

where the hydrolysis equivalent (*h*) is the amount of free amino groups produced during hydrolysis, expressed as millimoles of serine equivalents per gram protein and *h_tot_* is the total amount of amino groups present in totally hydrolysed casein with 6 N HCl at 110 °C for 24 h.

### 3.5. Size Exclusion High Performance Liquid Chromatography (SE-HPLC)

The average molecular weight (MW) of the casein hydrolysates was determined by SE–HPLC using a Shimadzu 10 AVP HPLC system equipped with a Biosuite 125/5 mm HR-SEC column (7.8 × 300 mm, Waters, Milford, MA, USA). Phosphate buffer (100 mM) containing 100 mM NaCl was used as the mobile phase at a flow rate of 0.5 mL/min at 25 °C. Protein in the effluent was monitored at 220 nm. A calibration curve was made from the log MW of the standard markers and their respective elution times (*R*^2^ = 0.99).

### 3.6. Matrix-Assisted Laser Desorption/Ionization–Time of Flight (MALDI–TOF) Spectrometry

MALDI–TOF–MS analysis was performed at the Alberta Proteomics and Mass Spectrometry facility (University of Alberta, AB, Canada). One microliter of each sample was mixed with 1 µL of sinapic acid (10 mg/mL in 50% acetonitrile/water + 0.1% trifluoroacetic acid) and was spotted onto a stainless steel target plate and allowed to air dry. All mass spectra were obtained using a Bruker Ultraflex MALDI–ToF/ToF (Bruker Daltonic, Bremen, Germany). Ions were analysed in the positive mode, and external calibration was performed by using a standard protein mixture.

### 3.7. Antioxidant Capacity Analysis

#### 3.7.1. 1,1-Diphenyl-2-picryl Hydrazyl (DPPH) Radical Scavenging Assay

The radical scavenging activity of casein hydrolysates at different concentrations (0.1, 1, and 5 mg/mL) was assessed according to the method described previously [58]. Ascorbic acid and GSH were used as positive controls. The radical scavenging activity of the hydrolysates was calculated according to the following equation:

Percentage DPPH free radical scavenging = 1 − (*A_s_*/*A_c_*) × 100 (2)

where *A_s_* and *A_c_* represent the absorbance of the sample and the control, respectively.

#### 3.7.2. Superoxide Radical Scavenging Assay

In the superoxide radical scavenging assay, 80 μL of casein hydrolysates (at 0.1, 1 and 5 mg/mL) was mixed with 80 μL of 50 mM Tris-HCl buffer (pH 8.3) in a 96-well microplate, followed by the addition of 40 μL of 1.5 mM pyrogallol in 10 mM HCl. The rate of superoxide-induced polymerization of pyrogallol (∆*A_s_*/min) was measured as the increase in absorbance at 320 nm for 5 min at 23 °C [59]. Glutathione was applied as a positive control and Tris-HCl buffer was used instead of hydrolysates in blank experiments (∆*A_c_*/min). The superoxide scavenging activity of the hydrolysates was calculated using the following equation:

Superoxide scavenging activity = [(*∆A_c_*/min − *∆A_s_*/min)]/(∆*A_c_*/min) × 100 (3)

#### 3.7.3. Iron Chelating Capacity

Ferrous ion chelating activity was determined according to the method of Zhang et al. [32]. EDTA, a strong metal chelator, was used as the positive control. Ferrous ion chelating ability was measured at different concentrations of casein hydrolysates (0.1, 1, and 5 mg/mL) and calculated by the following equation:

Percentage iron chelating capacity = 1 − (*B_s_*/*B_c_*) × 100 (4)

where *B_s_* and *B_c_* represent the absorbance of the sample and the control (deionized water instead of hydrolysates), respectively.

#### 3.7.4. Ferric Reducing Antioxidant Power (FRAP) Assay

The antioxidant capacity of the samples (at 0.1, 1, and 5 mg/mL) was estimated according to the procedure described by Fogarasi et al. [60]. Trolox (1–50 µM) was used as the positive control. The results were expressed as micromoles of Trolox equivalent (TE) per mg of sample (μmol TE/mg).

### 3.8. Anti-Inflammatory Properties of CH

#### 3.8.1. Cell Viability Assay

The proliferation of Raw 264.7 cells was evaluated using the 3-(4,5-dimethylthiazol-2-yl)-2,5-diphenyltetrazolium bromide (MTT) assay [61]. Briefly, cells were seeded in 96-well microplates at a density of 1 × 10^4^ cells/mL and incubated at 37 °C overnight. After treatment with casein hydrolysates (1 mg/mL) for 20 h and washing the cells with Hank’s buffered salt solution (HBSS), 100 μL of MTT reagent (0.5 mg/mL in media) was added and the cell was incubated in the dark for 4 h. Formazan crystals were solubilized in 200 μL dimethyl sulfoxide, and the color intensity was measured at 570 nm. The results were expressed as a percentage of the absorbance in the test wells compared to that in the control wells (media without peptide).

#### 3.8.2. Determination of Nitric Oxide (NO) Production (Griess Assay)

The effect of casein hydrolysates on NO production by stimulated macrophages was evaluated by the determination of accumulated nitrite (NO_2_^−^) in the culture media [62]. RAW 264.7 cells were cultured at 1 × 10^5^ cells/mL and incubated overnight. Cells were treated with casein hydrolysates (1 mg/mL) for 4 h. After washing cells with HBSS, bacterial LPS (1 μg/mL) was added to stimulate the macrophage cells and was incubated for 20 h. Then, 100 μL of cell supernatant was mixed (1:1 *v*/*v*) with the Griess reagent, followed by 15 min incubation at ambient temperature. The absorbance was recorded at 540 nm. The growing cells without any treatment acted as the negative control, while the positive controls were prepared by stimulating cells with LPS. Sodium nitrite at different concentration levels (0.1–100 μM) was used to make the standard curve (*R*^2^ = 0.9998).

#### 3.8.3. Real-Time Polymerase Chain Reaction (RT-PCR) Analysis for Cytokine Gene Expression

RAW 264.7 cells were plated in 6-wells at 5 × 10^4^ cells/cm^2^. After 24 h incubation at 37 °C, cells were treated using LPS (1 µg/mL) with casein and Fla-hydrolysed casein (10 µg/mL). Cells incubated in media served as the negative control. Cells treated only with LPS (1 µg/mL) were taken as the positive control. Glyceraldehyde 3-phosphate dehydrogenase (GAPDH) was used as the endogenous control. RNA was extracted by the TRIzol-chloroform method from RAW 264.7 cells stimulated with LPS (1 µg/mL) and then treated with Fla-treated casein (10 µg/mL) for 24 h. RNA purity was measured by a spectrophotometric method. The total RNA was reverse-transcripted into cDNA (1 μg of RNA sample in 20 µL of RT-reaction mixture) using the 5× All-In-One RT MasterMix for RT-PCR. qRT-PCR was performed in a Quantstudio III (Applied Biosystems, Carlsbad, CA, USA). The 10 µL qPCR reaction mixture consisted of 2.5 ng cDNA, 300 nM of each primer (tumor necrosis factor (TNF)-α, forward: 5′-TAC TGA ACT TCG GGG TGA TCG GTC C-3′, reverse: 5′-CAG CCT TGT CCC TTG AAG AGA ACC -3′; interleukin (IL)1β, forward: 5′-GGA GAA CCA AGC AAC GAC AAA ATA CC-3′, reverse: 5′-TGG GGA ACT CTG CAG ACT CAA AC-3′; GAPDH, forward: 5′-ACT TTG TAC AGC TCA TTT CC-3′, reverse: 5′-TGC AGC GAA CTT TAT TGA TG-3′) and 5 µL of EvaGreen 2× qPCR MasterMix-Low Rox. Water was used as the control. Each mRNA expression was normalized against GAPDH mRNA (ΔΔC_t_-method) and all data are presented as the fold change against the unstimulated control [63].

### 3.9. Liquid Chromatography-Tandem Mass Spectrometry (LC-MS/MS)

Hydrolysates were subjected to LC-MS/MS analysis on a nanoAcquity UPLC (Waters,) connected to a Q-TOF mass spectrometer equipped with an electrospray ionization source (Waters). Five microliters of the peptide were loaded onto a C_18_ PepMap 100 Nano-Precolumn (300 µm × 1 mm, Dionex, Sunnyvale, CA, USA) and nano analytical column (75 µm × 150 mm, C_18_ acclaim PepMap 100 column, Dionex). Desalting on the peptide trap was achieved by flushing trap with 1% acetonitrile and 0.1% formic acid in water, at a flow rate of 10 µL/min for 1 to 3 min. Peptides were separated with a gradient of 1–60% solvent B (acetonitrile, 0.1% formic acid) over 55 min, at a flow rate of 350 nL/min. The MS/MS data were analysed through proteomic software called Mascot software (version 2.2., Matrix Science, Boston, MA, USA). For the database search, the mass tolerance values for the parent ion and fragment ion were set at 0.1 and 0.2 Da, respectively. Up to two missed cleavages were selected. Oxidation on methionine was selected as the variable modification. Confidence of positive protein identification was judged by high protein and peptide scores in the search results. A manual inspection of the original MS/MS spectra was performed to assure that major peaks in the MS/MS spectra were matched and explained.

### 3.10. Statistical Analysis

All experiments were performed in at least three independent trials. The results are reported as means ± SD. The results were subjected to two-way analysis of variance followed by Duncan’s multiple range test using SAS software version 9.3 (SAS Institute, Cary, NC, USA). Statistical significance of differences was defined at the 5% level (*p* < 0.05).

## 4. Conclusions

Enzymatic hydrolysis of casein under high hydrostatic pressure (at 100 MPa for 1 h at E:S ratio of 1:50) improved the DH and resulted in hydrolysates with improved antioxidant properties compared to atmospheric pressure hydrolysis. The molecular weight distribution of hydrolysates reflected in SEC–HPLC and MALDI–TOF analysis confirmed that the HHP-EH method produces a greater proportion of short peptides compared to AP-EH. In general, high pressure processing significantly improved the DH, proportion of smaller peptides, and antioxidant properties of hydrolysates. The effect of high pressure was most notable on Try-hydrolysed casein, which showed a significant increase (4.1–26.5%) in the proportion of small peptides (500 up to >2000 Da), and showed an increased DPPH and superoxide radical scavenging capacity under HHP compared to AP. Overall, Fla hydrolysates resulted in higher DH and better antioxidant properties when considering the five enzymes tested. Casein hydrolysates were cyto-compatible and did not adversely influence macrophage cell growth. A significant reduction in the NO level after pre-incubation with casein hydrolysates indicates the inhibitory effect of the hydrolysates in inflammation. Therefore, the HHP-EH method provides a promising technology to produce bioactive peptides from various protein sources under mild hydrolysis conditions. In addition to the improved hydrolysis efficiency, HHP-EH is a simple, environmentally friendly, and economic alternative to conventional protein hydrolysis methods. The multifunctional peptides produced from casein through HHP-EH can be used in preparing therapeutic supplements or natural health products with antioxidant/anti-inflammatory properties, aiming to prevent/treat chronic diseases such as cardiovascular disease and cancers. Further investigations are required to study the in-depth structural characteristics of hydrolysates obtained from the HHP-EH digestion of proteins. Currently, we are working on the effect of HHP-EH on allergenicity and the functional properties of casein-derived peptides with regard to the structural changes which occur during the hydrolysis process.

## Figures and Tables

**Table 1:** Enzymes applied in high hydrostatic pressure combined with enzymatic hydrolysis (HHP-EH) and atmospheric hydrolysis, and the operational conditions.

Enzyme	Sources	Proteolytic Activity ^a^	Optimum Conditions
Elastase	Elastase from hog pancreas	≥4 U/mg	pH 8; 37 °C
Flavourzyme	Protease from *Aspergillus oryzae*	≥500 U/g	pH 7; 50 °C
Savinase	Protease from *Bacillus* sp.	≥16 U/g	pH 7; 55 °C
Thermolysin	Protease from *Bacillus thermoproteolyticus*	14 U/mg	pH 7; 50 °C
Trypsin	Protease derived from porcine pancreas	30 U/g	pH 7; 37 °C

^a^ Minimum proteolytic activity of the enzyme at optimum pH and temperature.

**Table 2:** Relative area (%) of the peptide peaks obtained in matrix-assisted laser desorption/ionization time-of-flight (MALDI–TOF) mass spectra of casein hydrolysates produced under an atmospheric and high pressure condition.

Samples	Atmospheric Hydrolysis			High Pressure Hydrolysis		
	500–1000 Da	1000–2000 Da	>2000 Da	500–1000 Da	1000–2000 Da	>2000 Da
Fla-CH	0.3	98.6	1.1	1.9	95.9	2.2
Try-CH	4.1	46.5	49.4	26.5	23.7	49.8

**Table 3:** Amino acid sequences of potent peptides in Fla-hydrolysed casein identified by LC–MS/MS and their respective protein fragments.

Peptide Sequence	Ion (*m*/*z*)	Observed Mass	Calculated Mass	Source (Fragment)
PGPIPN	594.33	593.33	593.32	β-Casein (78–83)
PFPGPIPN	838.44	837.43	837.44	β-Casein (76–83)
YPFPGPIP	887.47	886.47	886.46	β-Casein (75–82)
VYPFPGPIPN	1100.55	1099.55	1099.57	β-Casein (74–83)
MPFPKYPVEP	610.82 (2)	1219.62	1219.59	β-Casein (124–133)
EPVLGPVRGPFP	632.87 (2)	1263.73	1263.70	β-Casein (210–221)
QEPVLGPVRGPFP	696.90 (2)	1391.78	1391.76	β-Casein (209–221)
TPVVVPPFLQPE	661.87 (2)	1321.73	1321.72	β-Casein (95–106)
TQTPVVVPPFLQPE	776.42 (2)	1550.83	1550.83	β -Casein (93–106)
